# Immunotherapy-Induced Acute Tubulointerstitial Nephritis

**DOI:** 10.7759/cureus.15358

**Published:** 2021-05-31

**Authors:** Kevin Parza, Kevin Dao, Pooja Patel, Nicolina Scibelli, Andrew Mangano, Maryam Gondal

**Affiliations:** 1 Internal Medicine, Grand Strand Medical Center, Myrtle Beach, USA; 2 Nephrology, Grand Strand Medical Center, Myrtle Beach, USA

**Keywords:** cancer immunotherapy, nephrotoxicity, immune related adverse events, melanoma, nivolumab, ipilimumab, acute tubular interstitial nephritis, immune checkpoint inhibitor

## Abstract

Due to its minimal side-effect profile, immunotherapy has become a popular choice for the treatment of advanced melanoma as compared to conventional chemotherapy. The most common side effects associated with immunotherapy include gastrointestinal, pulmonary, and dermatologic manifestations. However, there have been very few documented occurrences of nephrotoxic side effects. We present a case of a 73-year-old male with a past medical history of chronic kidney disease (CKD) stage 3A, metastatic uveal melanoma, and gastroesophageal reflux disease on pantoprazole who arrived at the intensive care unit with altered mental status and creatinine of 27 gm/dl (baseline creatinine of 3 gm/dl about one year prior), after receiving his first dose of ipilimumab and nivolumab approximately 21 days prior. Kidney biopsy demonstrated acute tubulointerstitial nephritis (ATIN). This case highlights the importance of recognizing acute tubulointerstitial nephritis as a side effect of immunotherapy for prompt diagnosis and early treatment.

## Introduction

Ipilimumab and nivolumab are examples of novel immune checkpoint inhibitors (ICPi), which are selective antibodies used in the treatment of advanced melanoma. Multiple reports have shown that immunotherapy has a more favorable side-effect profile in comparison to general chemotherapy [1-2].

During an immune checkpoint interaction, a T-lymphocyte cell meets with an antigen-presenting cell (APC) to either activate or inhibit an inflammatory response. Cancer cells act as APCs and thus are allowed to proliferate if the programmed death (PD) pathway is activated. Ipilimumab is a cytotoxic T-lymphocyte-associated antigen 4 (CTLA-4) checkpoint inhibitor and nivolumab is a programmed death-1 (PD-1) checkpoint inhibitor. Together, these agents can ensure that tumor cells are likely to be recognized. As a result, T-lymphocyte cells are able to proliferate and initiate a proper immune response. Approximately, 60% to 85% of patients receiving ICPis may experience immunotherapy-related adverse effects (irAEs) due to a robust immune response [3]. The most common irAEs include dermatologic and gastrointestinal manifestations, with an incidence of 64.3% and 46.7%, respectively. Renal manifestations, however, are much less common, with an incidence of only 1.4% [3-4]. Checkpoint inhibitor-induced acute kidney injury (CPI-induced AKI) can present similarly to another medication-induced nephrotoxicity with mild proteinuria and elevated serum creatine [5]. A thorough workup is required to rule out other causes of AKI and establish the diagnosis of CPI-induced AKI.

## Case presentation

A 73-year-old male with a past medical history of metastatic uveal melanoma with liver metastasis, chronic kidney disease stage 3A, hypertension, type 2 diabetes, and hyperlipidemia presented with nausea, itchiness, and worsening confusion for three days. He had received his first dose of ipilimumab and nivolumab three weeks prior. He denied any chest pain, palpitations, abdominal pain, lightheadedness, anuria, and dysuria. The patient was seen at his nephrologist's clinic where laboratory data showed potassium of 8 mmol/L, creatinine of 2.7 mg/dl, glomerular filtration rate (GFR) of 2 mL/min/1.73 m, and ammonia level of 34 umol/L. Of note, the patient had a baseline creatinine of 3 gm/dl about one year prior to admission. He was directly admitted to the intensive care unit (ICU) for emergent dialysis. Urinalysis showed mild proteinuria of 200 mg/dl, urine glucose of 300 mg/dl, and was negative for nitrites, leukocyte esterase, and eosinophils. No urine casts were appreciated.

Upon admission to the intensive care unit (ICU), the patient’s vitals were stable, and he was started on appropriate hyperkalemic treatment consisting of calcium gluconate, polystyrene sulfonate, sodium bicarbonate, regular insulin, and dextrose. The nephrology service was consulted, and the patient was immediately started on oral prednisone 60 mg daily, as there was a strong suspicion of drug-induced nephropathy. A repeat basic metabolic panel (BMP) was repeated four hours later with no improvement. A triple lumen dialysis catheter was placed and the patient underwent emergent hemodialysis (HD).

**Figure 1:**
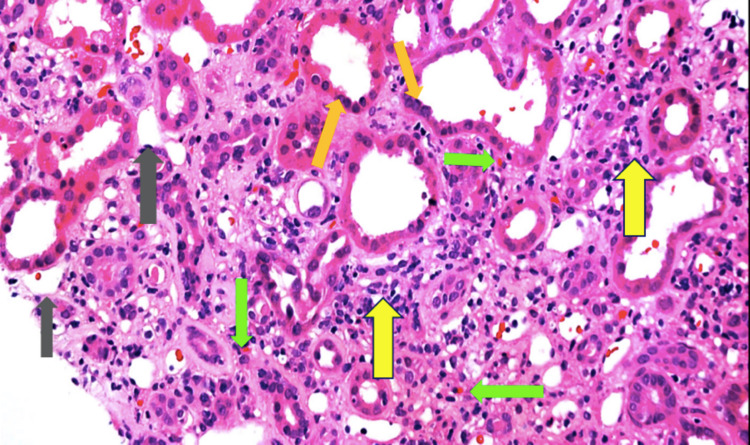
Kidney biopsy consistent with acute tubulointerstitial nephritis Lymphocytes in the tubular area (orange arrow). Lymphocytes and eosinophils in the interstitial area (yellow and green arrow). Interstitial edema (gray arrow).

A kidney biopsy was consistent with acute tubulointerstitial nephritis (ATIN) (Figure 1). Throughout the rest of his hospitalization, he had received a total of six rounds of hemodialysis until his discharge one week later. His creatinine had decreased to 11.60 mg/dl and his eGFR improved mildly to 4 mL/min/1.73m^2^. He was instructed to complete a steroid taper, stop immunotherapy, and was recommended to continue indefinite HD by his nephrologist.

## Discussion

While the precise pathophysiology of irAE is unknown, prior translational studies have suggested that autoreactive T-cells, pre-existing antibodies, and increased cytokine responses may contribute to the event [6]. Auto-reactive T-lymphocyte cells can increase in population during cross-reactivity with normal tissue antigens found in the myocardium in the heart, or melanocytes. Furthermore, pre-existing antibodies in patients with autoimmune diseases, such as thyroid disease, may increase the production of the same antibodies through the modulation of humoral immunity [3,7]. A research study focusing on colitis secondary to immunotherapy demonstrated an increased number of pro-inflammatory cytokines directly proportional to the number of T-lymphocytes. Immunoassays were obtained in the study to confirm that interleukin-17 (IL-17) levels were elevated compared to the baseline in the setting of colitis secondary to immunotherapy [6].

Most CPI-AKI presents similarly to other types of medication-induced AKI. Patients will often be asymptomatic but can also present with proteinuria, fever, rash, and hematuria [8]. Amongst the reported subtypes of CPI-AKI, acute tubulointerstitial nephritis (ATIN) was the most common with an average median time of development within three to six months of starting immunotherapy [8]. According to a recent prospective study, risk factors for CPI-AKI include pre-existing chronic kidney disease, concomitant proton pump inhibitor (PPI) use, and the administration of combination immunotherapy [3]. Prior epidemiologic studies note that several confounding variables, such as underlying cardiovascular disease, make it challenging to determine if CKD truly is an independent risk factor [9]. However, a multicenter study stated a strong association with patients on immunotherapy with underlying CKD developing an AKI [3].

Proton pump inhibitor-induced ATIN is rare. Interestingly, our patient was noted to be on omeprazole 20 mg daily upon admission. An epidemiologic study highlights that there is a greater likelihood when using immunotherapy concomitantly with proton pump inhibitors [10]. According to Shirali et al., the patients had improvement in CPI-AKI following the discontinuation of the proton pump inhibitor medication [11]. Larkin et al. conducted a randomized control trial on combination immunotherapy consisting of nivolumab and ipilimumab in the treatment of advanced melanoma. Findings included greater progression-free survivability with combination therapy than monotherapy alone at 11.5 months versus 2.9 months, respectively [12]. Despite these benefits, there is a correlation between combination immunotherapy use and increased nephrotoxicity (adjusted odds ratio, 3.88; 95% conﬁdence interval, 2.21 to 6.81) [3].

To evaluate for CPI-AKI, serial labs that can be obtained include serum creatinine, serum estimated glomerular filtration rate (eGFR), and urine analysis to monitor for proteinuria and eosinophiluria. A definitive diagnosis is obtained through a kidney biopsy [13]. Other differentials of acute kidney injury to consider in a cancer patient receiving immunotherapy include infection, dehydration, urinary tract obstruction, and any other medication-induced nephrotoxicity [8].

A grading system was established by the National Institute of Health (NIH) called the Common Terminology Criteria for Adverse Events (CTCAE), which encompasses grades 1-5. Mild adverse effects include grades 1-2 while grades 3-4 are classified as severe and grade 5 is associated with death [14]. In regards to the management of CPI-AKI, a thorough work-up is needed to ensure proper classification and to monitor for the advancement of the disease. Grade 1 patients have creatine levels >1.5 times the upper limit of normal. At this point, it is recommended to continue CPI therapy; however, one should discontinue all nephrotoxic agents. Grades 2 and 3 patients have serum creatinine that is >1.5 to 3 and >3.0 to 6 times the upper limit of normal, respectively. At these levels, hospitalization and nephrology consult are warranted. Nephrotoxic agents and CPI therapy are typically discontinued when concerns for CPI-AKI develop. At this point, patients are started on oral prednisone 1-2 mg/kg/day. Patients that fall into the grade 4 category have serum creatinine at least >6 times the upper limit of normal and should be considered for hemodialysis. Furthermore, these patients should be started on intravenous (IV) methylprednisolone and eventually transitioned to oral prednisone [14]. A multicenter study demonstrated improved outcomes with steroid treatment. In this study, 44% of the patients with CPI-AKI demonstrated complete recovery while only 16% showed complete recovery without steroids [3].

## Conclusions

While gastrointestinal, dermatologic, and pulmonary side effects are the most common manifestations of immune-related adverse effects, acute kidney injury can still occur. When dealing with patients on immunotherapy, especially those with underlying chronic kidney disease, clinicians should maintain a high index of suspicion for acute kidney injury secondary to immunotherapy. Specifically, acute renal failure in the setting of ipilimumab and nivolumab therapy should include acute tubulointerstitial nephritis in the differential. Proton pump therapy has also been associated with tubulointerstitial nephritis and concurrent usage with immunotherapy may increase this risk. Serial BMPs should be obtained frequently within the first weeks of infusion to monitor for acute kidney injury. If diagnosed with CPI-AKI, patients should promptly be started on steroids if indicated.
